# Misreporting of Patient Outcomes in the South African National HIV Treatment Database: Consequences for Programme Planning, Monitoring, and Evaluation

**DOI:** 10.3389/fpubh.2020.00100

**Published:** 2020-04-07

**Authors:** David Etoori, Alison Wringe, Chodziwadziwa Whiteson Kabudula, Jenny Renju, Brian Rice, F. Xavier Gomez-Olive, Georges Reniers

**Affiliations:** ^1^Department of Population Health, London School of Hygiene and Tropical Medicine, London, United Kingdom; ^2^MRC/WITS Rural Public Health Transitions Research Unit (Agincourt), Faculty of Health Sciences, School of Public Health, University of Witwatersrand, Johannesburg, South Africa; ^3^Kilimanjaro Christian Medical University College, Moshi, Tanzania; ^4^MeSH Consortium, Department of Public Health Environments and Society, Faculty of Public Health and Policy, London School of Hygiene and Tropical Medicine, London, United Kingdom

**Keywords:** HIV, retention in care, bias, South Africa, health information systems

## Abstract

**Background:** Monitoring progress toward global treatment targets using HIV programme data in sub-Saharan Africa has proved challenging. Constraints in routine data collection and reporting can lead to biased estimates of treatment outcomes. In 2010, South Africa introduced an electronic patient monitoring system for HIV patient visits, TIER.Net. We compare treatment status and outcomes recorded in TIER.Net to outcomes ascertained through detailed record review and tracing in order to assess discrepancies and biases in retention and mortality rates.

**Methods:** The Agincourt Health and Demographic Surveillance System (HDSS) in north-eastern South Africa is served by eight public primary healthcare facilities. Since 2014, HIV patient visits are logged electronically at these clinics, with patient records individually linked to their HDSS record. These data were used to generate a list of patients >90 days late for their last scheduled clinic visit and deemed lost to follow-up (LTFU). Patient outcomes were ascertained through a review of the TIER.Net database, physical patient files, registers kept by two non-government organizations that assist with patient tracing, cross-referencing with the HDSS records and supplementary physical tracing. Descriptive statistics were used to compare patient outcomes reported in TIER.Net to their outcome ascertained in the study.

**Results:** Of 1,074 patients that were eligible for this analysis, TIER.Net classified 533 (49.6%) as LTFU, 80 (7.4%) as deceased, and 186 (17.3%) as transferred out. TIER.Net misclassified 36% of patient outcomes, overestimating LTFU and underestimating mortality and transfers out. TIER.Net missed 40% of deaths and 43% of transfers out. Patients categorized as LTFU in TIER.Net were more likely to be misclassified than patients classified as deceased or transferred out.

**Discussion:** Misclassification of patient outcomes in TIER.Net has consequences for programme forecasting, monitoring and evaluation. Undocumented transfers accounted for the majority of misclassification, suggesting that the transfer process between clinics should be improved for more accurate reporting of patient outcomes. Processes that lead to correct classification of patient status including patient tracing should be strengthened. Clinics could cross-check all available data sources before classifying patients as LTFU. Programme evaluators and modelers could consider using correction factors to improve estimates of outcomes from TIER.Net.

## Introduction

At the end of 2017, it was estimated that there were 36.9 million people living with HIV (PLHIV) worldwide, with 70% of the disease burden situated in sub-Saharan Africa (1, 2). The World Health Organization's (WHO) revised HIV treatment guidelines in 2015 call for immediate provision of lifelong antiretroviral therapy (ART) to all people testing positive for HIV. By the end of 2017, 60% of PLHIV in sub-Saharan Africa were on ART (1, 2). Whilst ART initiation rates have been increasing over time, in order to reduce HIV transmission rates and achieve 90-90-90 AIDS elimination goals, there is a need for accelerated increases in treatment adherence and retention in care (3–5). South Africa has the largest population of PLHIV worldwide, with an estimated 18.8% of the adult population aged 15–49 years old living with HIV, representing 7.2 million people (1, 2). By the end of 2018, an estimated 68% of PLHIV in South Africa were on ART (1, 2).

The rapid growth in access to ART has accentuated the need for an affordable and accurate way to monitor and evaluate treatment programmes (6–8), including documenting the number of people alive and on treatment, and programme impact on mortality. In the past, the progress of patients on ART was mainly monitored through patient cohorts (9) and tallying numbers of services rendered to inform resource allocation (8). However, evaluation of HIV programmes has proved challenging due to multiple data constraints. These include concerns about data reliability (8), and continued use of paper registers which often lack unique identifiers, suffer from incompleteness (10), and are cumbersome to use with increasing patient numbers and length of patient follow-up (11, 12). Another major concern is “silent transfers” whereby patients change clinics informally and without accompanying documentation, a phenomenon which has become more prevalent with the expansion of ART programmes (13, 14). As a result, there is concern that many high-burden countries are ill-equipped to report on the outcomes of patients in care and on treatment (6, 7, 15–17).

In order to address these concerns, many countries are scaling-up the use of electronic patient registers (11). However, challenges persist including insufficient linkages between clinics (10), insufficient training of staff who are responsible for entering this information (10), and staff shortages (18–21), resulting in some staff responsible for data management being stretched across multiple roles (22). This sometimes leads to poor workflow, and staff resistance which results in poor change management. Privacy and security issues (22) are also a major concern.

In 2010, South Africa adopted TIER.Net, a three-tiered monitoring approach involving paper registers (TIER 1—recommended for facilities with <500 patients), an offline electronic register (TIER 2—recommended for facilities with 500–2,000 patients) and networked electronic medical records (TIER 3—recommended for facilities with more than 2,000 patients) (11). This allowed for different tiers to be implemented in each facility based on the context and resources available at the time of implementation and typically involved a phased evolution, beginning with preparation for TIER.Net, installation and training, back capturing, live capturing and finally a live site able to produce monthly and quarterly reports with staff on-site to manage it. In 2014, an estimated 3,000 out of 4,000 public sector clinics in South Africa were using TIER.Net (23, 24) in one of the three phases of implementation. As of 2017, TIER 3 was still in its pilot phase (25).

ART patient outcomes have evolved since the start of national HIV treatment programmes. In several cohort studies of ART programmes in sub-Saharan Africa, there have been reports of higher rates of LTFU among patients who initiated ART in later years compared to earlier years (26–28). This may be explained by patients increasingly initiating treatment while less severely ill (29), as well as a negative consequence of patient numbers increasing such as facility workloads (30), raising concerns about the sustainability of these programmes. Some systematic reviews have shown that the percentage of patients LTFU who have died has decreased in later years as eligibility criteria have evolved to include less immunologically compromised patients, and as the proportion of patients LTFU has increased (13, 31). Furthermore, scale-up and decentralization of these programmes means ART can be offered at clinics closer to patients' homes, which may serve as an incentive to self-transfer in order to continue treatment at more convenient locations (13, 32).

Unpublished TIER.Net analyses from 2018 showed LTFU rates to range from 11 to 15% in the first three months and from 27 to 34% in the first year of ART (Y.Pillay, HIV Think-tank update, March 19, 2019). The high percentages of LTFU present many issues. Firstly, if these patients have really stopped ART then they have a higher mortality risk (33–35), and are more likely to transmit HIV (36–39). Given that patients that are LTFU have poorer outcomes, LTFU can also through misclassification bias event rates such as mortality downwards (40), leading to biased performance indicators for ART programmes. Accurate mortality rates are also important as they are used as parameters for projections such as in the UNAIDS spectrum package (41, 42).

We compare patient outcomes recorded in TIER.Net to the outcomes ascertained through a record review and tracing study for patients deemed lost to follow-up in eight public sector health facilities in rural north-eastern South Africa. We aimed to assess misreporting in TIER.Net and potential biases in the national programme statistics reported from the TIER.Net database.

## Methods

### Setting

The Agincourt Health and Demographic Surveillance System (Agincourt HDSS) is located in rural north-eastern South Africa, in Mpumalanga province which has the second highest prevalence of HIV at 14.1% (43). HIV prevalence among people 15 years of age or older in the HDSS was estimated at 19.4% in 2010 (44). The Agincourt HDSS comprises of 31 villages covering an area of 475 square kilometers with an estimated population of 115,000 people (45, 46).

There are five primary health facilities and three secondary community health centers located within the Agincourt HDSS. Every HIV-positive patient has a clinical file that is opened when they first register at an ART clinic and updated at each clinic visit. Following the clinic visit, visit-level information from the patient file is entered into the national electronic database, TIER.Net. All health facilities routinely trace patients that are late for a scheduled clinic appointment. This tracing is done in conjunction with two non-profit organizations, Right to Care (RtC) and Home-Based Carers (HBC). Clinic staff must contact all patients first by phone and if this does not yield a satisfactory outcome, a home visit is organized. Patients are classified as lost to follow-up (LTFU) if they have not returned 90 days after their scheduled visit.

#### Demographic Surveillance

Data collection aims to capture all demographic events for the Agincourt HDSS population. Fertility, mortality and migration data are based on a comprehensive household registration system that has been in operation since 1992. Following the baseline demographic surveillance survey in 1992 and three update rounds until 1998, the site has conducted annual surveys since 1999 (45, 47–49). Trained fieldworkers visit each household and interview the most knowledgeable adult available. During the visit, individual-level information on all household members is checked and updated and any events that have occurred since the last census round are recorded. Starting in 2017, data have been collected utilizing an electronic data collection system using tablets (50).

#### Point-of-Contact Interactive Record Linkage (PIRL)

A key element of the data infrastructure for this study consists of HIV patient visit logs collected by a study fieldworker in the health facilities that provide ART in the area. This work started in April 2014 at seven government facilities and was extended in 2016 to include one additional health facility. In addition to logging patient visits, these records are linked to the Agincourt HDSS using a procedure that we have previously described as Point-of-Contact Interactive Record Linkage (PIRL) (51, 52). In brief, a fieldworker conducts a short uptake interview with patients in the waiting area of the clinic. Patients who consent are asked to declare a few personal identifiers that are used to search a local copy of the Agincourt HDSS database using a probabilistic algorithm. Matches are confirmed in interaction with the patients, and the names of other household members are used as a key attribute to adjudicate between possible matches.

### Record Review and Tracing Study

Through the PIRL database, we identified patients who were more than 90 days late for a scheduled clinic appointment from HIV services on August 15, 2017 (the date of data extraction) at any of the eight health facilities located in the Agincourt HDSS area. These patients were recruited into a cohort and followed up to ascertain their treatment and vital status i.e., whether they were still alive.

All PLHIV aged 18 years or over, who had ever declared residency in Agincourt HDSS, and had enrolled in the HIV treatment and care programme since 2014 (after the Agincourt HDSS record linkage was established at the health facilities) were eligible for inclusion in the study.

Trained and supervised fieldworkers conducted a thorough record review, comparing the list of patients LTFU against (a) TIER.Net (b) patient clinic files, and (c) logbooks kept by the RtC and the HBC. The PIRL database was also reviewed for duplicate patients (different clinical records linking to the same individual in the Agincourt HDSS database, which was taken as evidence of silent transfers), and residency and vital status were extracted from the Agincourt HDSS database. This was done on a case-by-case basis.

HBC conducted a visit to the households of all patients for whom a definitive outcome (defined as death, transfer out, stopped ART, migrated, re-engaged in care, and alive with ART status unknown) could not be established, or for whom routine patient tracing was not done. Finally, all patients who remained LTFU after the HBC visit, were searched for in TIER.Net databases at clinics in close proximity to their home residence to capture any further silent transfers.

We also reviewed the records for a stratified random sample of 162 patient records who were not LTFU as of August 15, 2017, in order to assess whether TIER.Net misclassified any patients that were still in care. This sample was chosen to include 18 patients from every clinic (six men, six non-pregnant women, and six women who initiated ART while pregnant) with the exception of one clinic which had recently merged with another, and from which we sampled 18 patients who had enrolled whilst in each of the clinics prior to the merger.

### Definitions

Definitions of terms used in this article are provided in Table 1.

**Table 1:** Definitions of terms used.

**Term**	**Definition**
The last appointment	The last scheduled appointment for each patient as of August 15, 2017, when we generated the list of patients deemed LTFU.
TIER.Net treatment status	The treatment status of the patient as recorded in TIER.Net during the comprehensive record review.
The final outcome	The outcome ascertained for each patient through the record review and tracing process.
Data error	A situation in which a patient was found still in care and <90 days late for their last appointment. Some data errors occurred because visit dates had not been properly entered in the PIRL database. Patients categorized as a data error were excluded from our analyses.
Re-engagement	A patient was considered to have re-engaged in care if they were found to be still in care at the same clinic where they initiated treatment but were >90 days late for their last appointment (had been LTFU).
Transfer out	A patient was considered to have transferred if they had either reported taking treatment at another clinic (for clinics outside the Agincourt HDSS), if their ART initiation clinic had communicated with and ascertained their transfer to another clinic, or if there was record of them collecting treatment at another clinic within the Agincourt HDSS.
Migration	A patient was classified as having migrated out of the study site if they were recorded as having migrated through the Agincourt HDSS demographic surveillance, this migration event happened after their last clinic visit date and there was no proof that they were taking treatment at another facility.
Alive and not on ART	A patient was considered alive and not on ART if they had been found and had said they had stopped ART, denied their HIV status or refused to return to the clinic.
Alive with ART status unknown	A patient was considered alive with ART status unknown if they were found to still be alive through the most recent Agincourt HDSS demographic surveillance, with a surveillance date after their last clinic visit and there was no proof that they were taking treatment at any facility.

#### Statistical Analyses

For patients included in the record review and tracing study, we calculated counts and proportions for socio-demographic and baseline clinical characteristics, TIER.Net treatment status, the final outcome, and cross-tabulated TIER.Net status and the final outcome.

To assess the degree and direction of mis-reporting of patient outcomes in TIER.Net, we graphically present TIER.Net treatment status and the final outcome proportions by some selected patient characteristics. A Pearson's chi-square test was used to compare whether TIER.Net treatment status and the final outcome varied by all the categorical variables. We also present a cross-tabulation of patient outcomes from the two sources.

A binary outcome variable was created to indicate whether TIER.Net had misclassified a patient's treatment status, with a second outcome created to identify whether the patient was recorded as LTFU in TIER.Net. All cases where an electronic record could not be found were removed from further analysis.

To explore factors associated with misclassification in TIER.Net, we ran bi-variate analyses with patient-level treatment characteristics, demographic characteristics and facility-level characteristics. All variables with *p* < 0.1 were included in the multivariable logistic regression model. A parsimonious model was achieved using Wald tests. This same procedure was followed in order to understand what factors were associated with being reported LTFU in TIER.Net. All analyses were conducted in Stata 15 (53) and all data visualization was done using R (54).

#### Ethics

Ethical approval was obtained from the London School of Hygiene and Tropical Medicine, the University of Witwatersrand and the Mpumalanga Department of health.

## Results

### Database Population Characteristics

Over the study period, 4,089 patients were added to the PIRL database and met the inclusion criteria. Of these 4,089, 1,325 (32.4%) met the LTFU criteria and were eligible for inclusion into the record review and tracing study. Of these 1,325 patients, 166 (12.5%) did not have an ART initiation date and were assumed to be pre-ART. Further investigation of these 166 patients found 46 (27.7%) had initiated ART after record linkage, 59 (35.5%) were genuine pre-ART patients and 61 (36.7%) had initiated ART before record linkage began. These 61 patients were excluded from further analyses. Of the remaining 1,264 patients, 190 (15.0%) were found to have data errors (mostly due to missing clinic visits in the PIRL database) and were excluded from further analysis (Supplementary Figure 1).

Of 1,074 remaining patients, 280 (26.1%) initiated ART for prevention of mother-to-child transmission (PMTCT), 737 (68.6%) met the ART initiation criteria for non-pregnant adults, and 57 (5.3%) had not initiated ART yet (pre-ART).

Thirteen (8.0%) of the 162 patients still in care were excluded from the analysis because they had not declared residency in the HDSS. The remaining 149 from the random sample of patients still in care were also assessed to see if misclassification also occurred for those who remained engaged in care (Table 2).

**Table 2:** Database population characteristics.

	**Not in sample**	**Sample**		
	**Still in care**	**Still in care**	**LTFU**	**Data error**
	**2,615**	**149**	**1,074**	**190**
	***N* (%)**	***N* (%)**	***N* (%)**	***N* (%)**
**SEX**				
Female	2,016 (77.1)	94 (63.1)	807 (75.1)	147 (77.4)
Male	599 (22.9)	55 (36.9)	266 (24.8)	42 (22.1)
Missing	0 (0)	0 (0)	1 (0.1)	1 (0.5)
**AGE**				
18–29	559 (21.4)	18 (12.1)	350 (32.6)	39 (20.5)
30–44	1,298 (49.6)	76 (51.0)	509 (47.4)	102 (53.7)
45–59	544 (20.8)	36 (24.2)	152 (14.1)	38 (20.0)
60+	212 (8.1)	19 (12.7)	60 (5.6)	10 (5.3)
Missing	2 (0.1)	0 (0)	3 (0.3)	1 (0.5)
**ART REASON**				
Non-PMTCT women	1,533 (58.6)	54 (36.2)	487 (45.3)	101 (53.1)
PMTCT women	431 (16.5)	40 (26.9)	280 (26.1)	45 (23.7)
Men	598 (22.9)	55 (36.9)	250 (23.3)	42 (22.1)
Pre-ART	0 (0)	0 (0)	57 (5.3)	2 (1.1)
Missing	53 (2.0)	0 (0)	0 (0)	0 (0)
**ART START YEAR**				
2014	320 (12.2)	62 (41.6)	211 (19.6)	41 (21.6)
2015	773 (29.6)	42 (28.2)	414 (38.6)	84 (44.2)
2016	951 (36.4)	32 (21.5)	350 (32.6)	54 (28.4)
2017	571 (21.8)	13 (8.7)	42 (3.9)	9 (4.7)
Missing	0 (0)	0 (0)	57 (5.3)	2 (1.1)
**Time ON ART**				
≤ 3 months	190 (7.3)	10 (6.7)	325 (30.3)	15 (7.9)
3–6 months	260 (9.9)	2 (1.3)	190 (17.7)	9 (4.7)
6–12 months	560 (21.4)	23 (15.4)	228 (21.2)	41 (21.6)
12–24 months	842 (32.2)	40 (26.8)	219 (20.4)	75 (39.5)
>24 months	763 (29.2)	74 (49.7)	55 (5.1)	48 (25.3)
Missing	0 (0)	0 (0)	57 (5.3)	2 (1.1)
**BASELINE CD4**				
<100	468 (17.9)	25 (16.8)	220 (20.5)	20 (10.5)
100–199	476 (18.2)	18 (12.1)	193 (18.0)	37 (19.5)
200–349	656 (25.1)	40 (26.9)	267 (24.9)	50 (26.3)
350–499	464 (17.7)	34 (22.8)	198 (18.4)	50 (26.3)
500+	455 (17.4)	30 920.1)	164 (15.3)	33 (17.4)
Missing	96 (3.7)	2 (1.3)	32 (3.0)	0 (0)
**REFILL SCHEDULE**				
1 month	1,056 (40.4)	38 (25.5)	714 (66.5)	72 (37.9)
2 months	1,016 (38.8)	77 (33.7)	240 (22.3)	64 (33.7)
3 months	314 (12.0)	26 (17.4)	86 (8.0)	17 (8.9)
>3 months	229 (8.8)	8 (5.4)	34 (3.2)	37 (19.5)
**HEALTH FACILITY**				
Agincourt	540 (20.6)	18 (12.1)	282 (26.3)	160 (84.2)
Belfast	379 (14.5)	16 (10.7)	191 (17.8)	2 (1.0)
Cunningmore	227 (8.7)	15 (10.1)	74 (6.9)	0 (0)
Justicia	284 (10.9)	18 (12.1)	122 (11.4)	3 (1.6)
Kildare	462 (17.7)	18 (12.1)	120 (11.1)	6 (3.2)
Lillydale/Bhubezi	487 (18.6)	35 (23.5)	181 (16.8)	11 (5.8)
Thulamahashe	89 (3.4)	14 (9.4)	27 (2.5)	8 (4.2)
Xanthia	147 (5.6)	15 (10.1)	77 (7.2)	0 (0)
**AGINCOURT HDSS OUTCOME**				
Still in HDSS	1,827 (69.9)	107 (71.8)	530 (49.3)	122 (64.2)
Deceased	2 (0.1)	0 (0)	83 (7.7)	1 (0.5)
Migrated	503 (19.2)	37 (24.8)	282 (26.3)	56 (29.5)
Not linked	283 (10.8)	5 (3.4)	179 (16.7)	11 (5.8)
**TIME SINCE LAST APPOINTMENT**				
≤ 1 year	–	–	539 (50.2)	–
1–2 years	–	–	392 (36.5)	–
>2 years	–	–	143 (13.3)	–

*Not in sample: All patients eligible for the study but not LTFU in the PIRL database and not included in the still in care sample; Sample: All patients included in the study (149 still in care, 1,264 LTFU = 1 074 really LTFU + 190 data errors); Data error: Patients included as LTFU but found to be still in care and <90 days late for their last appointment; For ART start year data from 2017 reflects number of ART initiations up to mid-August when data extraction occurred*.

### TIER.Net Treatment Status

Of the 1,074 patients who remained eligible for this analysis, 533 (49.6%) were categorized as LTFU, 222 (20.7%) as still in care, 186 (17.3%) as transferred out, 80 (7.5%) as deceased, and 53 (4.9%) could not be found in the TIER.Net database (Table 2).

There was a statistically significant difference (all *p* < 0.001) in the TIER.Net treatment status by sex, age, ART initiation status and reason, year of ART initiation, baseline CD4 count, time on ART, clinic visit schedule, health facility, and time since a missed appointment (see Supplementary Figure 2). Women who initiated ART for PMTCT were less likely to be categorized as deceased and more likely to be LTFU. All 149 patients sampled as still in care were also reported as still in care in TIER.Net.

### Outcomes After Record Review and Tracing Study

**Figure 1:**
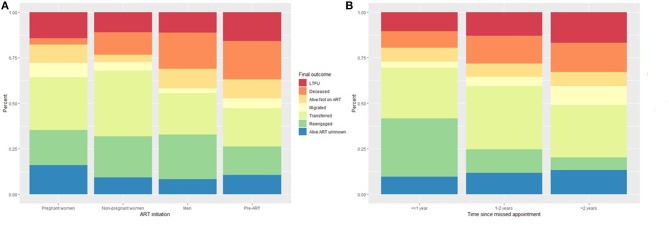
Outcome ascertained through record review and tracing stratified by **(A)** ART initiation status and **(B)** time since the last appointment.

Of the 1,074 patients who remained eligible for this analysis, 326 (30.3%) were found to have transferred to another clinic, 234 (21.8%) to have re-engaged in care, 132 (12.3%) were deceased, 117 (10.9%) were alive with ART status unknown, 81 (7.5%) were alive but not on treatment, 53 (4.9%) had migrated to another place of residence, and 131 (12.2%) were still LTFU (Table 3). These outcomes differed (all *p* < 0.001) by sex, age, ART initiation status and reason, baseline CD4 count, time on ART, clinic visit schedule, health facility, whether the patient record was successfully linked to an Agincourt HDSS record, and time since a missed appointment (some selected variables illustrated in Figure 1).

**Table 3:** A cross-tabulation of TIER.Net treatment status and the final outcome.

	**TIER.Net status**					
	**Deceased**	**Lost to follow-up**	**Not found**	**Still in care**	**Transferred out**	**Total**
**Final Outcome**	***N* (%)**	***N* (%)**	***N* (%)**	***N* (%)**	***N* (%)**	***N* (%)**
LTFU	0 (0)	112 (21.0)	9 (17.0)	10 (4.5)	0 (0)	131 (12.2)
Deceased	80 (100)	38 (7.1)	8 (15.1)	6 (2.7)	0 (0)	132 (12.3)
Alive/Not on ART	0 (0)	70 (13.1)	3 (5.7)	8 (3.6)	0 (0)	81 (7.5)
Migrated	0 (0)	47 (8.8)	5 (9.4)	1 (0.5)	0 (0)	53 (4.9)
Transferred	0 (0)	116 (21.8)	8 (15.1)	16 (7.2)	186 (100)	326 (30.4)
Re-engaged	0 (0)	56 (10.5)	9 (17.0)	169 (76.1)	0 (0)	234 (21.8)
Alive/ART unknown	0 (0)	94 (17.6)	11 (20.8)	12 (5.4)	0 (0)	117 (10.9)
Total	80	533	53	222	186	1074

### Differences Between TIER.Net Treatment Status and Final Outcomes

Records of deceased or transferred out patients documented in TIER.Net aligned with patients' final outcome (i.e., no inaccuracies found for these two statuses). However, TIER.Net misclassified 52 (39.4%) of 132 deaths. Of these 52, 38 (73.1%) were classified as LTFU, 6 (11.5%) as still in care, and 8 (15.4%) were not found in the system at all.

TIER.Net also misclassified 53 patients as still in care. Of these, 10 (18.9%) were found to be LTFU, 16 (30.2%) to have transferred, 12 (22.6%) as alive with unknown ART status, 8 (15.1%) alive but not on treatment, 6 (11.3%) to have died, and 1 (1.9%) to have migrated to another place of residence. TIER.Net correctly captured 186 (57.1%) of 326 transfers.

**Figure 2:**
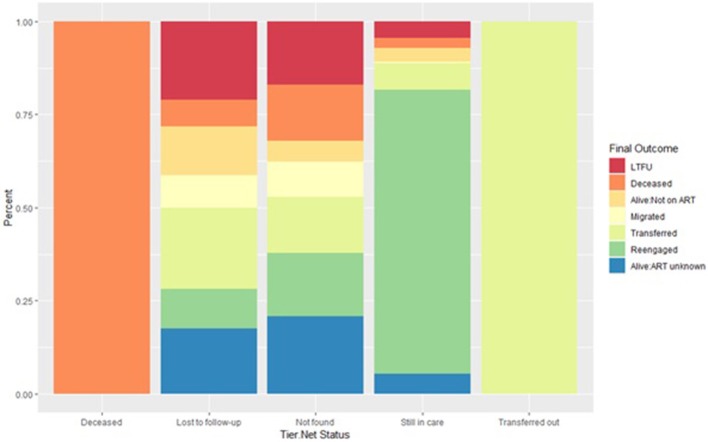
Outcome ascertained through record review and tracing stratified by each TIER.Net status. A deceased or transferred status in TIER.Net is accurate. Inaccuracies in the LTFU and still in care categories. Some patients categorized as still in care are LTFU, not currently on treatment, transfers out or dead.

Of 533 patients classified as LTFU by TIER.Net, 116 (21.8%) were found to have transferred to another clinic, 70 (13.1%) to be alive but not on treatment, 47 (8.8%) to have migrated to another place of residence, 38 (7.1%) to have died, and 56 (10.5%) to have re-engaged in care (38 of whom were resolved by new visit data in the PIRL database and so it is possible that their TIER.Net status could have also changed back to still in care) (Figure 2 and Supplementary Figure 3).

As patients classified as LTFU in TIER.Net were more likely to be misclassified we report on the factors associated with being classified as LTFU in TIER.Net (Supplementary Data and Supplementary Table 1).

### Factors Associated With Misclassification

In the multivariable model (Table 4), men (OR: 1.47, *p* = 0.021) had higher odds of misclassification when compared to women who initiated ART for non-PMTCT reasons (CD4, WHO stage, tuberculosis coinfection). Higher baseline CD4 (CD4 100-199 OR: 1.95. *p* = 0.002, CD4 ≥ 500 OR: 1.81, *p* = 0.014) was also associated with higher odds of misclassification when compared to patients who initiated treatment with CD4 <100. Health facility also remained statistically significant suggesting that facility level variability plays a role in misclassification. Patients who were linked to an Agincourt HDSS record in the PIRL database (OR: 2.09, *p* < 0.001) were more likely to be misclassified. Finally, patients who were between 1 and 2 years late (OR: 1.62, *p* = 0.001) were more likely to be misclassified. Older age (30–44 years OR: 0.73, *p* = 0.046, 45–59 years OR: 0.63, *p* = 0.046) was associated with lower odds of misclassification and patients on longer refill schedules (>3 months OR: 0.31, *p* = 0.009) were less likely to be misclassified.

**Table 4:** Factors associated with TIER.Net misclassification.

	**cOR (95%CI)**	***p*-value**	**aOR (95% CI) (*n* = 1,074)**	***p*-value**
**SEX**				
Female	Reference			
Male	1.08 (0.82–1.43)	0.584		
**AGE**				
18–29	Reference			
30–44	0.81 (0.62–1.07)	0.141	0.73 (0.54–0.99)	0.046
45–59	0.62 (0.41–0.92)	0.017	0.63 (0.40–0.98)	0.041
60+	0.72 (0.40–1.27)	0.253	0.70 (0.37–1.33)	0.279
**ART REASON**				
Non-PMTCT women	Reference		Reference	
Pregnant women	1.64 (1.22–2.20)	0.001	1.28 (0.92–1.78)	0.142
Men	1.34 (0.98–1.82)	0.063	1.47 (1.06–2.06)	0.021
Pre-ART	1.69 (0.83–3.47)	0.149	1.26 (0.57–2.78)	0.568
**ART START YEAR**				
2014	Reference			
2015	1.11 (0.79–1.54)	0.556		
2016	1.08 (0.76–1.52)	0.671		
2017	0.92 (0.47–1.80)	0.8		
**BASELINE CD4**				
<100	Reference		Reference	
100–199	1.83 (1.22–2.74)	0.003	1.95 (1.28–2.97)	0.002
200–349	1.29 (0.87–1.90)	0.201	1.45 (0.96–2.19)	0.074
350–499	1.32 (0.88–1.99)	0.183	1.52 (0.98–2.37)	0.063
≥500	1.62 (1.05–2.49)	0.029	1.81 (1.12–2.91)	0.014
**HEALTH FACILITY**				
Agincourt	Reference		Reference	
Belfast	2.42 (1.66–3.52)	<0.001	1.97 (1.30–2.97)	0.001
Cunningmore	2.01 (1.16–3.49)	0.013	1.64 (0.91–2.96)	0.101
Justicia	2.25 (1.46–3.47)	<0.001	1.96 (1.21–3.17)	0.006
Kildare	1.74 (1.12–2.71)	0.015	1.42 (0.89–2.28)	0.14
Lillydale/Bhubezi	2.53 (1.73–3.71)	<0.001	2.26 (1.50–3.41)	<0.001
Thulamahashe	1.14 (0.50–2.60)	0.762	1.47 (0.64–3.36)	0.363
Xanthia	1.74 (1.01–2.98)	0.045	1.86 (1.07–3.24)	0.028
**PIRL LINKAGE**				
Not linked	Reference		Reference	
Linked	1.59 (1.10–2.29)	0.014	2.09 (1.41–3.10)	<0.001
**TIME SINCE MISSED APPOINTMENT**				
<1 year	Reference		Reference	
1–2 years	1.89 (1.44–2.46)	<0.001	1.62 (1.21–2.17)	0.001
>2 years	1.58 (1.06–2.34)	0.025	1.34 (0.87–2.07)	0.181
**CLINIC VISIT SCHEDULE**				
1 month	Reference		Reference	
2 months	0.96 (0.72–1.28)	0.793	0.97 (0.71–1.34)	0.872
3 months	1.00 (0.64–1.57)	0.99	0.95 (0.59–1.52)	0.832
>3 months	0.19 (0.08–0.44)	<0.001	0.31 (0.13–0.74)	0.009
**TIME ON ART**				
≤ 3 months	Reference			
3–6 months	0.83 (0.57–1.20)	0.314		
6–12 months	0.72 (0.51–1.01)	0.055		
12–24 months	0.59 (0.42–0.83)	0.003		
>24 months	0.32 (0.18–0.56)	<0.001		

## Discussion

In this paper, we described the discrepancies between the treatment, vital and residency status of HIV patients enrolled in care between April 2014 and August 2017 in a rural South African setting as recorded in the national treatment database (TIER.Net) and their treatment outcome following a comprehensive record review and tracing study.

We found that TIER.Net misclassified 36% of the patient outcomes. ART initiation reason, baseline CD4, health facility attended, PIRL linkage, time since the last appointment, age, and ART refill schedule were all found to be significantly associated with misclassification. TIER.Net underestimated mortality and overestimated the number of patients who were LTFU. Seventy-nine percent of patients classified as LTFU in TIER.Net had a final outcome ascertained, mirroring findings from a systematic review of low and middle income country ART programmes which found that tracing generated higher estimates of mortality and lower estimates of LTFU (55). Our findings show that LTFU is still an important problem in ART programmes in this setting, even with routine patient tracing in place. TIER.Net also missed 43% of transfers with these silent transfers being the biggest contributor to misclassification among those documented as LTFU. We also found that 21.8% of patients had re-engaged in care, a phenomenon that was previously not well understood, but which is now increasingly recognized as becoming a common feature of ART programmes (55). Using our findings to revise LTFU figures to reflect re-engagement may help improve programme evaluation and forecasting.

In our study, we found that 40% of deaths were missed by TIER.Net, indicating that mortality of ART patients would be underestimated if relying on this data source. Given the role that national statistics play in HIV/AIDS projections (41, 42, 56), our findings suggest a need for correction factors for the estimates of the effect of ART on mortality. Although South Africa has a good vital registration system in place (57), these data are not currently linked to clinic-based information. However, with the move to registering patient national ID numbers, clinics should consider matching patients that are LTFU to the national death registry and other available databases such as the national health laboratory services database to ascertain vital status as this has proved useful in other studies in South Africa (58–60). Clinics in the Agincourt HDSS study area and other HDSS sites could also consider using vital status data from annual demographic surveillance to ascertain vital status for all patients.

The number of patient transfers to another clinic that were missed in TIER.Net suggests that communication between clinics is sub-optimal and that the current system for transferring patients between clinics can be improved. With studies reporting patient fear and concern about provider reactions if they return to care after a treatment interruption (61, 62) it is possible that some patients considered it less stressful to self-transfer or restart treatment at another nearby clinic, rather than returning to the facility where they had initiated treatment. These silent transfers could lead to double counting of patients currently on treatment, the second of the 90-90-90 targets, potentially suggesting that the programme is performing better than it is. Furthermore, given that the national treatment programme relies on data from TIER.Net to plan and procure ART based on active patient numbers, misclassification in the database, and more specifically double-counting due to silent transfers may lead to inaccurate drug forecasts and misestimation of medicines and other commodities at the national level. This bias will only increase as the South African ART programme expands with more patients potentially moving into new clinics closer to their homes and more people initiate ART with the move to test and treat. Future work will consider how application of correction factors from this research would change programme statistics and drug forecasts.

It is also important to consider the risk that silent transfers pose with regards to drug resistance, as this misclassifies treatment experienced patients as treatment naïve and may lead to patients being offered regimens that have lost their optimal therapeutic benefit. This is particularly concerning because resistance testing is not commonly used in these settings, and can potentially lead to increases in levels of transmitted drug resistance (63). Better referral systems, patient education, regular information exchange between clinics, and provider training (64), could improve recording of transfers and clinic staff attitudes toward less adherent patients. The WHO also recommends enforcement of unique identifiers as paramount to improve patient safety, improve the efficient use of programme resources by reducing duplications, and to improve programme monitoring and evaluation (65). With national IDs becoming mandatory at clinic registration, information exchange could prove useful in identifying silent transfers. This should also become less of an issue when TIER.Net is upgraded to a fully networked database.

We found several factors to be associated with misclassification of outcome in TIER.Net, with older age and longer ART refill schedules found to be protective factors. Older patients were less likely to be classified as LTFU in TIER.Net which probably explains why they were subsequently less likely to be misclassified. Given that longer ART refill schedules are synonymous with previous good adherence (66), these patients accounted for 11% of the patients LTFU and were also more likely to have re-engaged in care, a category that contributed very little to misclassification. Patients whose clinic record was successfully linked to an Agincourt HDSS record in the PIRL database were more likely to be misclassified. They were also more likely to have been resolved which could explain this association. Health facilities were also positively associated with misclassification, with the facilities with the highest proportion of patients classified as LTFU in TIER.Net being more likely to misclassify patients. Two of these clinics also had issues with routine tracing, with one clinic not undertaking any physical tracing at all, emphasizing an additional benefit of routine tracing. Finally, patients who had been LTFU for a longer duration were more likely to be classified as LTFU in TIER.Net, more likely to have transferred to another clinic, and less likely to have re-engaged in care which probably explains their higher likelihood of misclassification.

This analysis has several limitations. Firstly, TIER.Net was only consulted at a specific point in time. The cross-sectional nature of TIER.Net outcomes means that some may have changed, but we would have no way to ascertain this. We checked TIER.Net 12 months after the initial record review for all patients whose outcome after record review and tracing was still LTFU and 85% of the outcomes had not changed. However, for patients whose final outcome was resolved through new visit data in the PIRL database, it is likely that their TIER.Net outcome also changed. It is also possible that some of the patients categorized as LTFU in TIER.Net are due to the rigidity of the system as TIER.Net only allows for four possible outcomes; still in care, transferred out, LTFU and deceased (11, 67, 68). It is possible that for some patients, their outcomes were ascertained, but the rigidity meant that they could not be recorded in the database and may call for the inclusion of other possible outcomes in the database. The exclusion of patients for whom an electronic record could not be found from the multivariable analyses might bias our findings. However, given the relatively small number we expect that this bias is fairly small. Finally, we did not adjudicate causes of death, so it is possible that patients died from causes other than those related to HIV/AIDS. A strength of this study is that we attempted to trace all patients that were LTFU and not a sample. Therefore, the findings might be more generalizable to other settings. The multiple methods, data sources and levels of follow-up used to trace patients are also a strength.

In conclusion, although TIER.Net misclassified 36% of patient outcomes, this reflects the various challenges with the processes and upstream factors that lead to this misclassification and calls for their improvement rather than the utility of the database itself, as patients classified as LTFU were most likely to be misclassified. Clinics should consider training staff about ascertaining patient outcomes, putting more emphasis into patient tracing and using other data sources such as the national death register to improve ascertainment of patient treatment outcomes. For policy and planning purposes, programme evaluators should consider using correction factors to improve the accuracy of estimates from TIER.Net.

## Data Availability Statement

The data used in these analyses are not yet publicly available as they currently being utilized for the first author's Ph.D. research. They will be deposited with the Agincourt HDSS data manager and made available on request at the end of his Ph.D. in 2021. Data from the PIRL database are also available by making a data request to the Agincourt HDSS data manager.

## Ethics Statement

Ethical approval was obtained from the London School of Hygiene and Tropical Medicine, the University of Witwatersrand and the Mpumalanga Department of health.

## Author Contributions

The study was conceived by DE, AW, and GR. Fieldwork was planned and executed by DE, FG-O, and CK. Data collection was supervised by DE. Analyses were conducted by DE with input from all authors. The manuscript was drafted by DE with input from BR, JR and all the other authors. All authors contributed to the interpretation of the findings and read and approved the final manuscript.

### Conflict of Interest

The authors declare that the research was conducted in the absence of any commercial or financial relationships that could be construed as a potential conflict of interest. The reviewer PA declared a shared affiliation, with no collaboration, with the authors to the handling editor at the time of review.
