# Supplementary material for: Misreporting of Patient Outcomes in the South African National HIV Treatment Database: Consequences for Programme Planning, Monitoring, and Evaluation
Source: Front Public Health. 2020 Apr 7;8:100. doi: 10.3389/fpubh.2020.00100 (PMC7154050; doi:10.3389/fpubh.2020.00100)
Supplement: Supplementary file 1 [file Data_Sheet_1.docx]

Supplementary Material

# Supplementary Data: Factors associated with being categorised as LTFU in TIER.Net

In the multivariable model (Supplementary Table 1), initiating ART for PMTCT (OR: 1.69, p=0.004) and baseline CD4 between 100-349 (CD4 100-199 OR: 1.76, p=0.012, CD4 200-349 OR: 1.72, p=0.01) were associated with higher odds of being categorised as LTFU in TIER.Net. Older age (age 30-44 OR; 0.71, p=0.042), later ART initiation date (2016 OR: 0.43, p<0.001, 2017 OR: 0.12, p<0.001), and longer time on ART (6-12 months OR: 0.61, p=0.015, 12-24 months OR:0.35, p<0.001, >24 months OR: 0.20, p<0.001) were associated with lower odds of being categorised as LTFU in TIER.Net. Likelihood of being categorised as LTFU also varied by health facility with Belfast (OR: 3.11, p<0.001), Justicia (OR: 2.54, p<0.001) and Bhubezi (OR: 3.82, p<0.001) more likely to classify patients as LTFU when compared to Agincourt clinic.

# Supplementary Figures and Tables

## Supplementary Figures

190 patients categorized as data errors

61 patients who initiated ART before record linkage began

1074 patients LTFU included in final analysis

1264 patients presumed LTFU

1325 patients presumed LTFU (166 had no ART initiation date)

4089 patients aged 18+ years, declared residency in Agincourt (3923 initiated ART after record linkage began, 166 with no ART initiation date)

**Supplementary Figure 1.** Numbers of patients that were eligible at each stage and the number of patients excluded at each stage


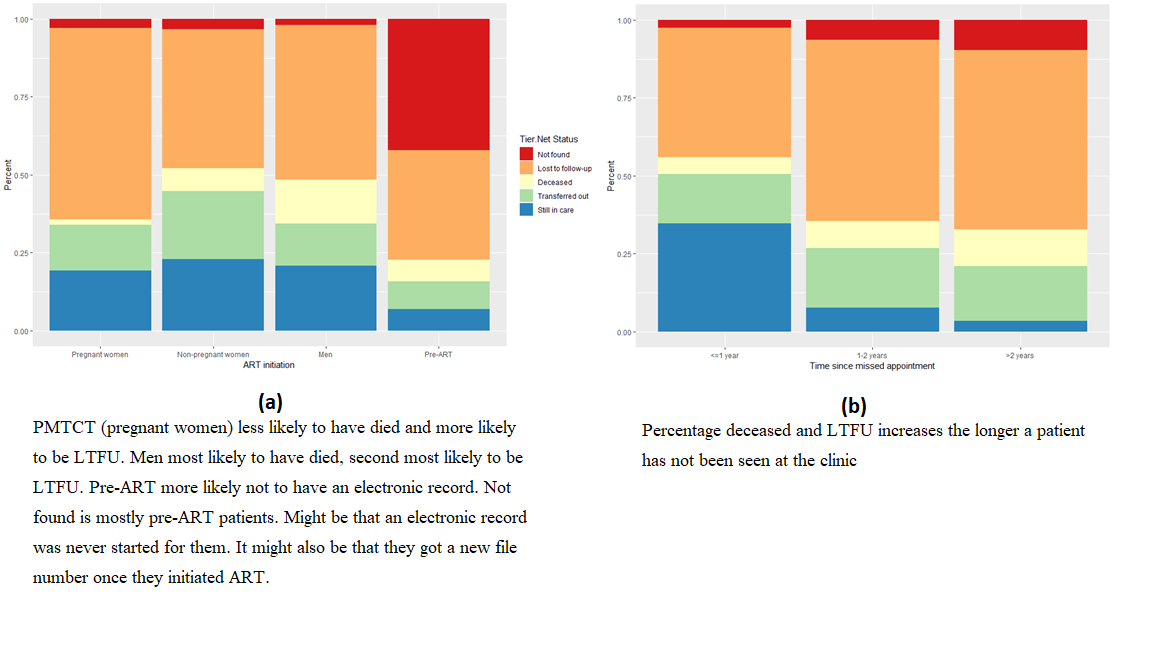


**Supplementary Figure 2.** TIER.Net treatment status by (a) ART initiation status and (b) time since the last clinic appointment


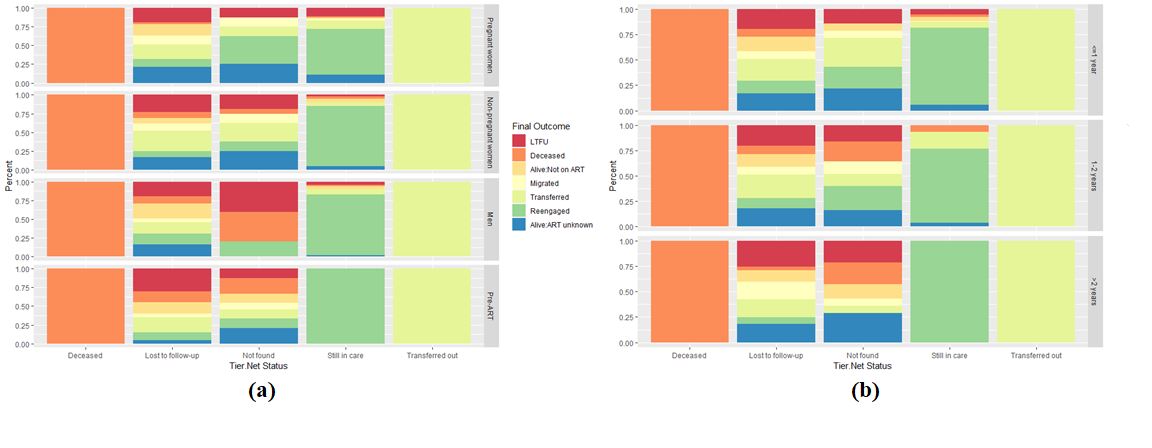


**Supplementary Figure 3.** Final outcome by TIER.Net treatment status and (a) ART initiation status and (b) time since the last clinic appointment

## Supplementary Tables

**Supplementary Table 1:** Factors associated with being classified as LTFU in TIER.Net.

|  | cOR (95%CI) | p-value | aOR (95% CI) (n=963) | p-value |
| --- | --- | --- | --- | --- |
| Sex |  |  |  |  |
| Female | Reference |  |  |  |
| Male | 0.92 (0.69 to 1.22) | 0.554 |  |  |
| Age |  |  |  |  |
| 18-29 | Reference |  | Reference |  |
| 30-44 | 0.81 (0.61 to 1.07) | 0.143 | 0.71 (0.51 to 0.99) | 0.042 |
| 45-59 | 0.63 (0.42 to 0.93) | 0.021 | 0.64 (0.39 to 1.03) | 0.065 |
| 60+ | 0.50 (0.28 to 0.89) | 0.019 | 0.53 (0.28 to 1.02) | 0.059 |
| ART reason |  |  |  |  |
| Non-PMTCT women | Reference |  | Reference |  |
| Pregnant women | 2.01 (1.48 to 2.73) | <0.001 | 1.69 (1.18 to 2.43) | 0.004 |
| Men | 1.20 (0.88 to 1.63) | 0.249 | 1.24 (0.88 to 1.76) | 0.219 |
| Pre-ART | 1.80 (0.88 to 3.70) | 0.11 | __ | __ |
| ART start year |  |  |  |  |
| 2014 | Reference |  | Reference |  |
| 2015 | 1.01 (0.71 to 1.41) | 0.967 | 0.77 (0.51 to 1.16) | 0.209 |
| 2016 | 0.98 (0.69 to 1.38) | 0.892 | 0.43 (0.27 to 0.68) | <0.001 |
| 2017 | 0.41 (0.20 to 0.84) | 0.015 | 0.12 (0.05 to 0.31) | <0.001 |
| Baseline CD4 |  |  |  |  |
| <100 | Reference |  | Reference |  |
| 100-199 | 1.73 (1.16 to 2.58) | 0.007 | 1.76 (1.13 to 2.73) | 0.012 |
| 200-349 | 1.70 (1.17 to 2.45) | 0.005 | 1.72 (1.14 to 2.59) | 0.01 |
| 350-499 | 1.31 (0.88 to 1.94) | 0.179 | 1.22 (0.77 to 1.93) | 0.4 |
| >=500 | 1.14 (0.74 to 1.73) | 0.552 | 0.99 (0.60 to 1.64) | 0.96 |
| Health Facility |  |  |  |  |
| Agincourt | Reference |  | Reference |  |
| Belfast | 3.28 (2.21 to 4.86) | <0.001 | 3.11 (2.02 to 4.79) | <0.001 |
| Cunningmore | 2.00 (1.17 to 3.43) | 0.012 | 1.64 (0.89 to 3.05) | 0.114 |
| Justicia | 2.90 (1.86 to 4.52) | <0.001 | 2.54 (1.56 to 4.13) | <0.001 |
| Kildare | 1.63 (1.05 to 2.53) | 0.029 | 1.56 (0.97 to 2.50) | 0.067 |
| Lillydale/Bhubezi | 3.79 (2.51 to 5.72) | <0.001 | 3.82 (2.40 to 6.06) | <0.001 |
| Thulamahashe | 1.11 (0.48 to 2.55) | 0.805 | 0.90 (0.35 to 2.34) | 0.83 |
| Xanthia | 1.06 (0.62 to 1.80) | 0.834 | 1.00 (0.56 to 1.77) | 0.987 |
| PIRL linkage |  |  |  |  |
| Not linked | Reference |  |  |  |
| Linked | 0.81 (0.58 to 1.13) | 0.218 |  |  |
| Time since missed appointment |  |  |  |  |
| < 1 year | Reference |  |  |  |
| 1-2 years | 2.22 (1.69 to 2.92) | <0.001 |  |  |
| >2 years | 2.36 (1.59 to 3.52) | <0.001 |  |  |
| Clinic visit schedule |  |  |  |  |
| 1 month | Reference |  |  |  |
| 2 months | 1.14 (0.84 to 1.53) | 0.394 |  |  |
| 3 months | 0.90 (0.57 to 1.44) | 0.669 |  |  |
| >3 months | 0.30 (0.13 to 0.68) | 0.004 |  |  |
| Time on ART |  |  |  |  |
| <=3 months | Reference |  | Reference |  |
| 3-6 months | 1.19 (0.81 to 1.72) | 0.373 | 0.91 (0.60 to 1.39) | 0.661 |
| 6-12 months | 0.75 (0.53 to 1.06) | 0.108 | 0.61 (0.41 to 0.91) | 0.015 |
| 12-24 months | 0.43 (0.30 to 0.62) | <0.001 | 0.35 (0.22 to 0.54) | <0.001 |
| >24 months | 0.30 (0.16 to 0.57) | <0.001 | 0.20 (0.09 to 0.43) | <0.001 |
